# Dr. William S. Ely

**Published:** 1911-02

**Authors:** 


Dr. William S. Ely, of Rochester, N. Y., died at his home January 11, 1911, of angina pectoris consecutive to influenza, aged 70 years. He was born in Rochester and lived there all his life, receiving his academic education at Rochester University, taking his A.B. degree in 1860. The next year the civil war broke out and he took service as assistant surgeon of the 108th volunteer infantry. Later he was promoted and, finally, became second in command at Annapolis General Hospital, one of the larger and more important of the army general hospitals.

Returning from army service and after receiving his medical degree, Dr. Ely began practice in earnest, following closely in the footsteps of his illustrious father who was an eminent physician. He soon attracted attention as a diagnostician of great acumen and his opinion was sought far and wide. He rose rapidly to eminence and within a few years stood at the forefront of his profession. He was attending physician to the Rochester City Hospital for many years. He was president of the Medical Society of the State of New York in 1887. In 1891 he was appointed a member of the New York State Board of Medical Examiners, serving as such until his death, being vice-president of the board.

Dr. Ely was a great physician; besides, too, he was an eminent citizen, finding time in the midst of an exacting practice to interest himself in the affairs of the city and to work for municipal betterment. He is survived by a wife and a son, who will receive the sympathy of a sorrowing community.

